# PARP1 negatively regulates transcription of BLM through its interaction with HSP90AB1 in prostate cancer

**DOI:** 10.1186/s12967-023-04288-z

**Published:** 2023-07-06

**Authors:** Mengqiu Huang, Lin Chen, Yingchu Guo, Yong Ruan, Houqiang Xu

**Affiliations:** 1 Laboratory of Animal Genetics, Breeding and Reproduction in the Plateau Mountainous Region (Ministry of Education), College of Life Sciences, Guizhou University, Guiyang 550025 Guizhou, China; 2 College of Animal Science, Guizhou University, Guiyang 550025 Guizhou, China; 3 Department of Ophthalmology, Affiliated Hospital of Zunyi Medical University, Zunyi 563000 Guizhou, China; 4 Guizhou University school of Medicine, Guizhou University, Guiyang 550025 Guizhou, China

**Keywords:** BLM, PARP1, ML216, Olaparib, Prostate cancer

## Abstract

**Background:**

Prostate cancer (PCa) is a prevalent malignant disease affecting a significant number of males globally. Elevated expression of the Bloom’s syndrome protein (BLM) helicase has emerged as a promising cancer biomarker, being associated with the onset and progression of PCa. Nevertheless, the precise molecular mechanisms governing BLM regulation in PCa remain elusive.

**Methods:**

The expression of BLM in human specimens was analyzed using immnohistochemistry (IHC). A 5′-biotin-labeled DNA probe containing the promoter region of BLM was synthesized to pull down BLM promoter-binding proteins. Functional studies were conducted using a range of assays, including CCK-8, EdU incorporation, clone formation, wound scratch, transwell migration, alkaline comet assay, xenograft mouse model, and H&E staining. Mechanistic studies were carried out using various techniques, including streptavidin-agarose-mediated DNA pull-down, mass spectrometry (MS), immunofluorescence (IF), dual luciferase reporter assay system, RT-qPCR, ChIP-qPCR, co-immunoprecipitation (co-IP), and western blot.

**Results:**

The results revealed significant upregulation of BLM in human PCa tissues, and its overexpression was associated with an unfavorable prognosis in PCa patients. Increased BLM expression showed significant correlations with advanced clinical stage (*P* = 0.022) and Gleason grade (*P* = 0.006). In vitro experiments demonstrated that BLM knockdown exerted inhibitory effects on cell proliferation, clone formation, invasion, and migration. Furthermore, PARP1 (poly (ADP-ribose) polymerase 1) was identified as a BLM promoter-binding protein. Further investigations revealed that the downregulation of PARP1 led to increased BLM promoter activity and expression, while the overexpression of PARP1 exerted opposite effects. Through mechanistic studies, we elucidated that the interaction between PARP1 and HSP90AB1 (heat shock protein alpha family class B) enhanced the transcriptional regulation of BLM by counteracting the inhibitory influence of PARP1 on BLM. Furthermore, the combination treatment of olaparib with ML216 demonstrated enhanced inhibitory effects on cell proliferation, clone formation, invasion, and migration. It also induced more severe DNA damage in vitro and exhibited superior inhibitory effects on the proliferation of PC3 xenograft tumors in vivo.

**Conclusions:**

The results of this study underscore the significance of BLM overexpression as a prognostic biomarker for PCa, while also demonstrating the negative regulatory impact of PARP1 on BLM transcription. The concurrent targeting of BLM and PARP1 emerges as a promising therapeutic approach for PCa treatment, holding potential clinical significance.

## Background

PCa is the second most common malignancy among men, representing 7% of newly diagnosed cancers in men worldwide. Annually, there are nearly 1.2 million new cases of PCa and approximately 350, 000 deaths related to the disease, making it one of the leading causes of cancer-related deaths in men [1]. Genetic alterations in basal or luminal prostate epithelial cells are considered the primary cause of the disease [2]. Androgen deprivation therapy (ADT) is recognized as the primary treatment for PCa due to its ability to reduce androgen receptor (AR) signaling. This therapy involves the use of chemical or surgical castration to lower androgen levels in order to suppress tumor growth and progression [3]. Despite the effectiveness of initial therapy, PCa inevitably progresses to castration-resistant prostate cancer (CRPC). CRPC can be further classified into non-metastatic castration-resistant prostate cancer (nmCRPC) and metastatic castration-resistant prostate cancer (mCRPC), based on the presence or absence of metastasis [4–6]. The administration of two AR-targeted drugs, abiraterone and enzalutamide, has shown notable extension in overall survival among patients with CRPC [4, 7]. However, resistance to these drugs can develop in certain patients, highlighting the need for further investigation into the underlying mechanisms of primary and acquired resistance. Understanding these mechanisms is crucial for guiding future treatment decisions [8]. Therefore, there is an urgent need for more effective and targeted therapeutic regimens in the management of CRPC.

Bloom’s syndrome protein (BLM) is a 3′-5′ ATP-dependent RecQ DNA helicase, playing a crucial role as one of the most essential genome stabilizers. It is involved in the regulation of DNA replication, recombination, and both homologous and non-homologous pathways of double-strand break (DSB) repair [9–11]. In silico analysis of The Cancer Genome Atlas (TCGA) datasets was conducted, revealing that BLM mRNA is overexpressed in cancer tissues compared to normal tissues [12]. In addition, a family-based study on PCa and a genome-wide haplotype association research conducted in the Chinese population have identified certain risk variants associated with the BLM gene in PCa. According to the study, the BLM gene is ranked highest among the seven identified high-risk PCa genes [13]. There have been limited studies on inhibitors targeting BLM helicase, and currently, ML216 is the only small molecule inhibitor commercially available for use [10, 14]. ML216 is a selective inhibitor of tumor cell growth that effectively impairs the function of BLM in human cells. It achieves this by competitively inhibiting the DNA binding activity of BLM [15]. By effectively suppressing the DNA unwinding activity of BLM, the inhibition induced by ML216 results in an elevated occurrence of sister chromatid exchange [16, 17]. A study has demonstrated the association of BLM with replication stress and drug resistance in multiple myeloma cells. The research findings propose BLM as a novel therapeutic target for the treatment of multiple myeloma [18]. Furthermore, studies have revealed that the inhibition of BLM can synergize with poly (ADP-ribose) polymerase (PARP) inhibitors, such as olaparib, a PARP inhibitor (PARPi). This combination has shown the ability to enhance the radiosensitivity of olaparib-resistant non-small cell lung cancer cells by inhibiting the homologous recombination repair (HRR) process [19]. While ML216 has demonstrated promising potential in tumor research, further investigation is warranted to elucidate its precise mechanism of action and explore strategies to enhance its safety and stability for achieving a more effective anticancer effect.

PARP1 is a well-known ADP-ribosylating enzyme that is activated upon binding to DNA single-strand breaks and DSBs [20, 21]. In addition to its pivotal role in DNA repair, PARP1 is known to engage in diverse cellular processes. It actively participates in chromatin remodeling, transcriptional regulation, and modulation of intricate cellular signaling pathways. PARP1 exhibits intricate interactions with an array of transcription factors and co-regulators, thereby exerting influence over gene expression profiles and modulating cellular responses to environmental stimuli [22, 23]. Aberrant PARP1 activity has been associated with the development and progression of cancer. PARP1 inhibitors, such as olaparib [24], is utilized for the treatment of tumors with DNA repair defects, particularly those caused by BRCA mutations. It has already received FDA approval for the treatment of advanced ovarian cancer and CRPC associated with defective BRCA genes [25]. However, several studies have indicated that certain BRCA-mutant cells may exhibit resistance to PARPi [26, 27]. Identifying non-BRCA-mutant patient subgroups that could still respond favorably to PARPi and preventing the development of drug resistance are therefore two major clinical challenges associated with the use of olaparib.

In this study, we confirmed the high expression of BLM at both the mRNA and protein levels in PCa and identified PARP1 as a negative transcriptional regulator of BLM in PC3 cells. Additionally, we demonstrated that the transcriptional regulation of BLM is enhanced by HSP90AB1 through its interaction with PARP1, counteracting PARP1’s negative impact on BLM. The functional experiments revealed that the combination therapy of ML216 and olaparib exhibited superior effects compared to monotherapy, both in vitro and in vivo.

## Materials and methods

### Data resources

Transcript data for BLM in PRAD was obtained from TCGA projects. The freely accessible GEPIA tool (http://gepia2.cancer-pku.cn/) was employed for genetic association analysis to examine patient outcomes. Disease-free survival (DFS) was evaluated using Kaplan–Meier curves, where different levels of BLM expression were assessed using the GEPIA tool. This tool integrates information from the Genotype Tissue Expression (GTEx) and TCGA projects.

### Cell culture and drugs

The RWPE-1, LNCaP, 22RV1, PC3, and DU145 cell lines were obtained from Zhong Qiao Xin Zhou Biotechnology Co., Ltd. (Shanghai, China). HEK-293T cells were purchased from Kunming Cell Bank (Kunming, China). The cells were cultured and aliquots were stored in liquid nitrogen for future use. RWPE-1 cells were cultured in customized medium (ZQXZ Bio, Shanghai, China). LNCaP, 22RV1, and DU145 cells were cultured in RPMI-1640 medium supplemented with 10% fetal bovine serum (FBS) and 1% penicillin–streptomycin (P/S) at 37 °C in a 5% CO_2_ environment. PC3 cells were cultured in ATCC-formulated F-12K medium supplemented with 10% FBS and 1% P/S at 37 °C in a 5% CO_2_ environment. HEK-293T cells were cultured in DMEM/HIGH GLUCOSE (HyClone, Logan, Utah, USA) supplemented with 10% FBS and 1% P/S at 37 °C in a 5% CO_2_ environment. Olaparib (AZD2281) and ML216 (CID-49852229) were purchased from MedChemExpress (Monmouth Junction, NJ, USA).

### Antibodies

The BLM antibody (bs-12872R) was procured from Bioss (Beijing, China). Antibodies against HSP90AB1 (11405-1-AP), XRCC4 (15817-1-AP), and GAPDH (60004-1-Ig) were obtained from Proteintech (Wuhan, China). Antibodies against PARP1 (ab227244), γH2AX (phospho S139) (ab81299), ATM (ab32420), ATM (phospho S1981) (ab81292), RPA70 (ab79398), Mre11 (ab109623), DNA-PKcs (ab32566), DNA-PKcs (phospho S2056) (ab124918), and Ki-67 (ab92742) were purchased from Abcam (Cambridge, UK). The antibody against 53BP1 (DF7472) was acquired from Affinity (Jiangsu, China).

### Plasmids and short hairpin RNA transfection

The expression plasmids PARP1oe and HSP90AB1oe, containing human PARP1 and HSP90AB1, respectively, were cloned into the pcDNA3.1-RFP-hismyc vector. Different fragments of the BLM promoter region were cloned into the pGL4.10-basic vector. The sequences of short hairpin RNA (shRNA) targeting PARP1 and HSP90AB1 were provided in Table 1. All shRNAs were synthesized by GenePharma. Transfection of all plasmids was performed using FuGENE®HD transfection reagent (Promega Corporation, Madison, WI, USA).

**Table 1:** The sequences of short hairpin RNA

Target gene	Sequence (5′–3′)
shPARP1-1	CACCGCAAAGGCCAGGATGGAATTGTTCAAGAGACAATTCCATCCTGGCCTTTGCTTTTTTG
shPARP1-2	CACCGGAGTCAAGAGTGAAGGAAAGTTCAAGAGACTTTCCTTCACTCTTGACTCCTTTTTTG
shPARP1-3	CACCGGACCAAGTGTATGGTCAAGATTCAAGAGATCTTGACCATACACTTGGTCCTTTTTTG
shPARP1-4	CACCGCTGTGAACTCCTCTGCTTCATTCAAGAGATGAAGCAGAGGAGTTCACAGCTTTTTTG
shHSP90AB1-1	CACCGAGGCTATCCCATCACCCTTTATTCAAGAGATAAAGGGTGATGGGATAGCCTTTTTTTG
shHSP90AB1-2	CACCGACAGCTGTGATGAGTTGATATTCAAGAGATATCAACTCATCACAGCTGTCTTTTTTG
shHSP90AB1-3	CACCGAAGTCCATCTATTACATCACTTTCAAGAGAAGTGATGTAATAGATGGACTTTTTTTTG
shHSP90AB1-4	CACCGAGAAGGTGACAATCTCCAATTTCAAGAGAATTGGAGATTGTCACCTTCTCTTTTTTG

### Western blot analysis

Cells were collected, and proteins were extracted using RIPA lysis buffer. The protein concentrations in the lysates were determined using the BCA method. Equal amounts of protein from each sample were mixed with SDS loading buffer, resolved by SDS-PAGE, and transferred to polyvinylidene difluoride (PVDF) membranes. After blocking with nonfat milk at 37 °C for 2 h, the membranes were incubated with the primary antibody overnight at 4 °C. Subsequently, the membranes were incubated with the appropriate secondary antibody at a 1:10,000 dilution for 1 h at 37 °C. After washing with TBST, the blot was incubated with an enhanced chemiluminescence detection reagent.

### RNA extration and RT-qPCR assay

Total RNA was extracted using the Trizol method (TaKaRa, JAPAN). For quantitative RT-qPCR analysis, RNA reverse transcription was performed using the PrimeScript RT reagent kit (TaKaRa, JAPAN). The amplification reaction, with a total volume of 40 μL, included 1.7 μL of both the sense and antisense primers, 1.6 μL of the cDNA template, 13 μL of ddH_2_O, and 22 μL of the DNA polymerase SsoFast EvaGreen Supermix (Bio-Rad, Hercules, California, USA). The mixture was vortexed and shaken, and a fluorescent polymerase chain reaction detection system (Bio-Rad, Hercules, California, USA) was used. The abundance of mRNA was determined using the 2−ΔΔCt method. The primers used are listed in Table 2.

**Table 2:** The primers sequences of RT-qPCR

Target gene	Sequence (5′–3′)
PARP1	F-TGGAAAAGTCCCACACTGGTA, R-AAGCTCAGAGAACCCATCCAC
BLM	F-AAGCGACATCAGGAGCCAAT, R-GAAGAACTATCACCCCCCAGC
HSP90AB1	F-TTGACATCATCCCCAACCCTC, R-ACCAAACTGCCCAATCATGGA
GAPDH	F-AAATCCCATCACCATCT, R-CCCCAGCCTTCTCCAT

### Polymerase chain reaction (PCR) assay

The PCR reaction mixture was prepared by combining 25 μL of PCR 2× Hieff Canace® Plus PCR Master Mix (Yeason, Shanghai, China), 2 μL of forward primer (10 μM), 2 μL of reverse primer (10 μM), 1 μL of template DNA, and sterile distilled water to a final volume of 50 μL. The reaction mixture was gently mixed and placed in a thermal cycler. The PCR cycling conditions were as follows: initial denaturation at 98 °C for 3 min, followed by 35 cycles of denaturation at 98 °C for 10 s, annealing at 60 °C for 20 s, and extension at 72 °C for 30 s. A final extension step was performed at 72 °C for 5 min. The PCR products were analyzed by agarose gel electrophoresis and visualized by ethidium bromide staining. The primers used are listed in Table 3.

**Table 3:** The primers sequences of PCR

Target gene	Sequence (5′–3′)
BLM-1	F-ACGAGCTCTACAAAAAGCTAGCTGGGCATGAT, R-CCGCTCGAGAAACATCAGTCTCTACTGAAATCA
BLM-2	F-CGAGCTCTACAAAAAGCTAGCTGGGCATGAT, R-CCGCTCGAGAAACATCAGTCTCTACTGAAATCA
BLM-3	F-CTGAGCTCTACAAAAAGCTAGCTGGGCATGAT,R-TCCTCGAGAAACATCAGTCTCTACTGAAATCAAATTTAC
BLM-4	F-GAGCTCTACAAAAAGCTAGCTGGGCATGATG, R-CTCGAGAAACATCAGTCTCTACTGAAATCAAATTTACC

### Streptavidin-agarose pull-down assay

A biotin-labeled double-stranded oligonucleotide probe of the BLM promoter sequence was synthesized by Genecreate Co. (Wuhan, China). Briefly, 500 μg of nuclear protein extract was mixed and incubated with 5 μg of the probe and 100 μL of streptavidin-agarose beads (Beaver Biosciences, Jiangsu, China) at 4 °C overnight. The mixtures were then centrifuged at 5000×*g*, resuspended in 30 μL of loading buffer, and boiled at 100 °C for 10 min. The collected samples containing the bound proteins were separated by SDS-PAGE for further silver staining or western blot analysis.

### Silver staining and MS

After electrophoresis of the samples containing the bound proteins, the protein gel was immersed in a solution of 10% acetic acid, 50% ethanol, and 40% water at room temperature on a shaker overnight. The protein bands were visualized using a fast silver staining kit (Solarbio, Beijing, China) and subsequently analyzed using MS by Genecreate (Wuhan, China).

### Dual luciferase reporter assay

Cells were seeded in 24-well plates and transfected with 0.5 μg/well of luciferase reporter plasmids. To normalize the transfection efficiency, the cells were co-transfected with 10 ng of pRL-TK (Renilla luciferase) plasmid. After 48 h of transfection, luciferase activity was detected using the Dual Luciferase Reporter Assay System Kit (E1910; Promega, Madison, WI, USA) following the manufacturer's instructions.

### Co-IP

PC3 cells cultured in a 25 cm^2^ cell culture flask were lysed on ice for 10 min using IP Binding buffer and PMSF. To investigate the interaction between endogenous PARP1 and HSP90AB1, the supernatants of cell lysates were incubated with protein A/G beads (BeaverBio, Suzhou, China) overnight at 4 °C. Prior to this, the protein A/G beads had been incubated with antibodies for 15 min at room temperature on a rotating wheel. For immunoprecipitation (IP), the supernatants of cell lysates were incubated with antibodies against PARP1 or IgG at 4 °C overnight. The beads were then washed with IP washing buffer and add 25 µL of 1× SDS-PAGE Loading Buffer to each tube. Mix thoroughly and heat at 95 °C for 5 min. Subsequently, conduct magnetic separation to collect the supernatant. For SDS-PAGE analysis, the input samples were loaded at 20% of the total volume, whereas the IgG and IP samples were loaded at 100%. Then transfer the proteins to PVDF membranes for further analysis.

### ChIP-qPCR assay

The ChIP assay was conducted using the SimpleChIP® Plus Enzymatic Chromatin IP Kit (9005, Cell Signaling Technology) following the manufacturer’s instructions. Briefly, cells were cross-linked with 1% formaldehyde, and the chromatin was sheared by sonication to generate DNA fragments of approximately 200–500 bp. The fragmented chromatin was immunoprecipitated using specific antibodies against PARP1 or HSP90AB1. After washing, the protein-DNA complexes were eluted from the beads, and the cross-linking was reversed by heating. The purified DNA was then subjected to qPCR analysis using primers specific to the genomic region of BLM. In the ChIP-qPCR experiment, the % Input value is calculated using the formula:

$$\Delta {\text{Ct}}\;\left[ {{\text{normalized}}\;{\text{ChIP}}} \right] = {\text{Ct}}\;\left[ {{\text{ChIP}}} \right] - \left( {{\text{Ct}}\;\left[ {{\text{Input}}} \right] - {\text{Log}}2\;\left( {{\text{Input}}\;{\text{Dilution}}\;{\text{Factor}}} \right)} \right)$$

$$\% \;{\text{Input}} = 2\left( { - \Delta {\text{Ct}}\;\left[ {{\text{normalized}}\;{\text{ChIP}}} \right]} \right) \times 100\% .$$

The primer sequences were listed in Table 4.

**Table 4:** The primers sequences of ChIP-qPCR

Target gene	Sequence (5′–3′)
BLM-1	F-TGAGAGTCCCATTTCTGCATCT, R-TGTTCAAGTTACCTCCCTCCT
BLM-2	F-TGCTCCTTGAAGACATTATGCT, R-CTGTAAGTGCGAGGTACCG
BLM-3	F-CCCACTTTCCCGGTTCAATG, R-TCTCTGTTTCACCCGTACCC
BLM-4	F-ACCGTCGGAACCAAGAGAAT, R-TTGAGAGCTGAGACTTGCCA

### IF and IHC

Cells were initially seeded onto coverslips in a 6-well plate. Subsequently, the cells were fixed with 4% paraformaldehyde for 15 min, permeabilized with 0.5% Triton-X for 5 min, and then blocked with 5% bovine serum albumin (BSA) for 30 min. The coverslips were then incubated with primary antibodies overnight at a dilution of 1:200. After washing, the coverslips were incubated with secondary antibodies at room temperature for 30 min and stained with 4,6-diamidino-2-phenylindole. The IF images were captured using an Olympus IX71 Nikon imaging system. The tissue chips containing PCa tissue samples and adjacent normal tissue samples from 90 patients were purchased from Super Biotek Inc. For IHC staining, the chips were incubated overnight at 4 °C with a primary antibody against BLM. Following washing with phosphate-buffered saline (PBS), the chips were incubated with a secondary antibody conjugated to a detection system. The sections were then developed using a diaminobenzidine substrate and counterstained with hematoxylin. The protein expression was visualized under a light microscope. Two independent observers, who were blinded to the experimental conditions, assessed the proportion of positively stained cells and the intensity of the IHC signal in the tumor sections. The staining intensity was scored on a scale of 0 to 3, with 0 indicating negative staining, 1 indicating weak staining (light yellow), 2 indicating moderate staining (yellow–brown), and 3 indicating strong staining (brown). The staining index (SI) was calculated by multiplying the proportion score by the staining intensity score.

### Cell viability assay, EdU assay and clony formation assay

Cell viability was determined using the Cell Counting Kit-8 (CCK-8) from APExBIO Technology LLC (Houston, USA). The cells, treated with drugs or transfected with different plasmids, were seeded at a density of approximately 4 × 10^3^ cells per well in 96-well plates and incubated in medium containing either a single drug or a combination of drugs. At 24 h, 48 h, and 72 h of incubation, the medium was supplemented with 10% CCK-8 and incubated for an additional 2 h. Cell viability was measured at 450 nm using a Multiskan Spectrum instrument (Synergy H4, BioTek, USA).

To assess cell proliferation status following different treatments, an EdU assay was performed according to the instructions provided in the manual (APExBIO Technology LLC, Houston, USA).

For the clony formation assay, cells were seeded at a density of 500 cells per well in 6-well plates and cultured for 2 weeks. The resulting colonies were fixed with formalin and stained with crystal violet. The number of colonies was counted using ImageJ 1.8.0 software (National Institutes of Health, Bethesda, MD, USA).

### Wound scratch assay and transwell assay

For the scratch wound healing assay, cells were cultured in 6-well plates until reaching confluence. A scratch was made using a 10 μL pipette tip, and the gap widths at 0 h (w1) and at 24 or 48 h (w2) were measured. The relative migration rate was calculated using the formula: (w1 − w2)/w1 × 100%.

In the Transwell assay, BD BioCoat 9 Matrigel Invasion Chambers (BD Biosciences, Franklin Lakes, NJ, USA) were utilized. Matrigel was diluted with DMEM/F12 medium and added to the upper chambers, which were then incubated at 37 °C for 6 h. Single-cell suspensions were added to the upper chambers coated with Matrigel. After 48 h, the invaded cells on the bottom of the chambers were stained with crystal violet and counted in five random fields.

### Alkaline comet assay

After a 48-h drug treatment, the cells were harvested and embedded in agarose on a microscope slide. Subsequently, the slides were treated with lysis buffer to remove cellular proteins and expose the DNA. The DNA was then subjected to electrophoresis under alkaline conditions, enabling the migration of DNA fragments according to size. Following electrophoresis, the slides were washed and stained with propidium iodide dye. The extent of DNA damage was visualized using a fluorescence microscope, and the comet tail length and intensity were scored to assess the level of DNA damage.

### In vivo xenograft studies

Male BALB/c nude mice aged 5–6 weeks were obtained from SJA Laboratory Animal Co., Ltd. (Changsha, China) and housed in quarantine for 1 week prior to experimentation. To establish tumor xenografts, 1 × 10^6^ PC3 cells suspended in 100 μL of PBS were injected into the right flank of the nude mice. The animals were then randomly divided into treatment and control groups, each containing 6 mice. The mice in the treatment groups received intraperitoneal injections of olaparib (30 mg/kg) and ML216 (15 mg/kg), either alone or in combination, three times a week for a duration of 3 weeks. The tumor volume was assessed every 4 days throughout the experimental period. Tumor volume was calculated using the formula (length × width^2^)/2. All animal experimental procedures were conducted in accordance with the guidelines and regulations set forth by the Laboratory Animal Ethics Committee of Guizhou University, and the study protocol was approved by the committee.

### Statistical analysis

The data were presented as the mean ± standard deviation (SD) from a minimum of three independent experiments. Statistical significance between two groups was determined using Student’s two-tailed t-test, while one-way ANOVA was used for multiple group comparisons. All statistical analyses were conducted using GraphPad Prism 8.0. A two-sided P-value of less than 0.05 was considered statistically significant (*P < 0.05, **P < 0.01, ***P < 0.001, ****P < 0.0001), while "ns" denotes no significant difference.

## Results

### BLM overexpression is a poor prognostic biomarker for PCa patients, and BLM knockdown inhibited cell proliferation and migration in vitro

**Fig. 1:**
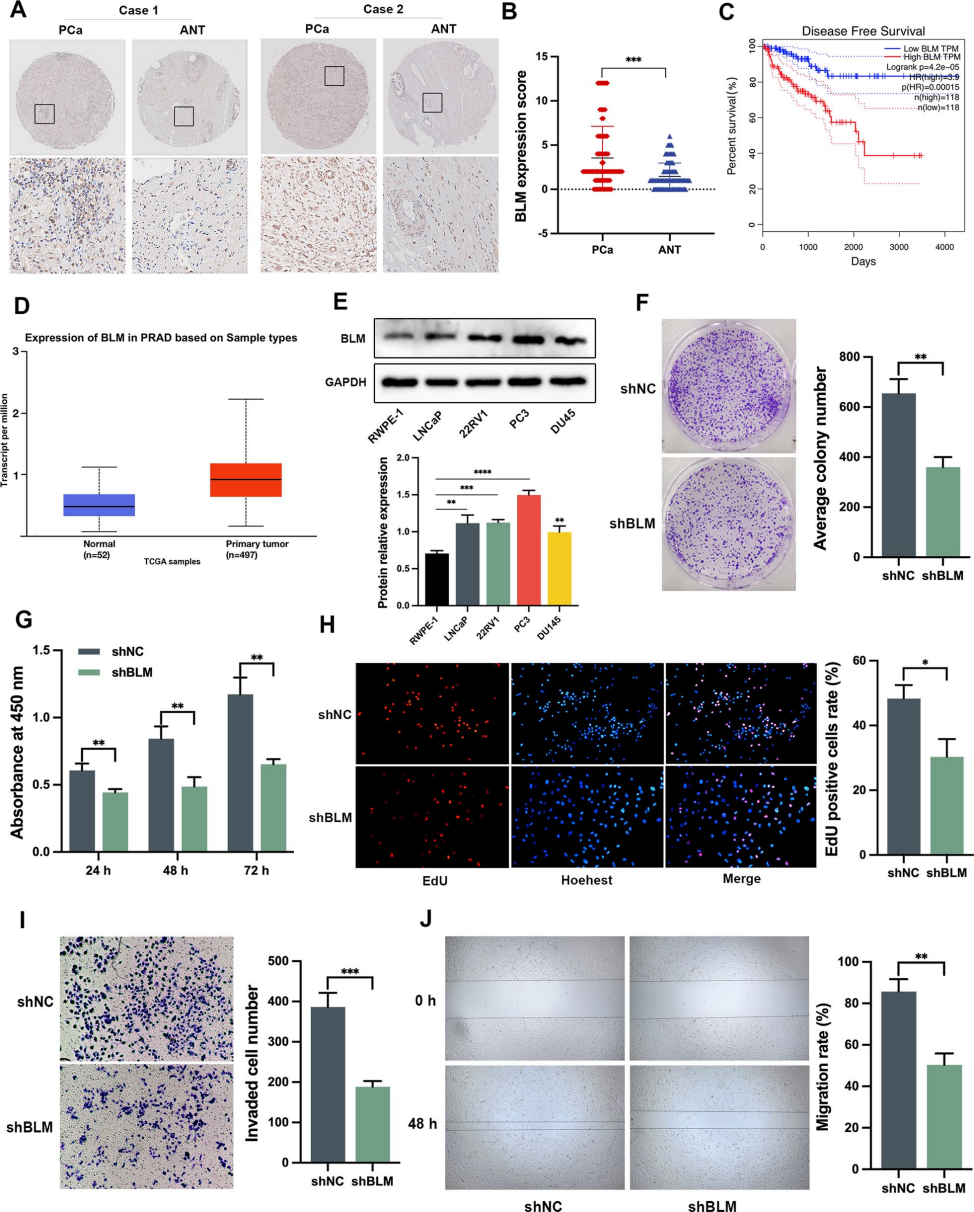
BLM serves as a prognostic biomarker for PCa, and knockdown of BLM has been shown to inhibit cell proliferation and migration in PC3. **A** Representative images demonstrating BLM expression in PCa tissues and ANT. The scale bars represent 500 μm and 100 μm. **B** The expression of BLM was higher in 90 PCa tissues compared to ANT. **C** High expression of BLM was associated with poor DFS in PCa patients. **D** Transcript levels of *BLM* in prostate adenocarcinoma (PRAD) as obtained from the TCGA database. **E** Western blot analysis of BLM in PCa cell lines (LNCaP, 22RV1, PC3, and DU145) and human normal prostate epithelial cells (RWPE-1). **F**–**J** Knockdown of BLM inhibited the clonogenicity, vitality, proliferation, invasion, and migration of PC3 cells. Shown are the means ± SD from 3 experiments (*P < 0.05, **P < 0.01, ***P < 0.001, ****P < 0.0001, and “ns” indicated no significant difference)

We performed IHC analysis on 90 paraffin-embedded PCa tumor tissues and their adjacent normal tissues (ANT) to evaluate BLM expression. Our results revealed that BLM expression was significantly higher in PCa tissues compared to ANT, with predominant localization in the nucleus (Fig. 1A, B). Furthermore, we examined the correlation between BLM expression and various clinicopathological characteristics of PCa patients, including age, clinical stage, Gleason score, Gleason grade, N-regional lymph nodes, and M-distant metastasis. The results, as shown in Table 5, demonstrated a significant association between BLM expression and clinical stage (χ^2^ = 2.288, *P* = 0.022) as well as Gleason grade (χ^2^ = 2.743, *P* = 0.006). However, no significant correlations were observed between BLM expression and age, N-regional lymph nodes, and M-distant metastasis (*P* > 0.05). Additionally, our analysis using GEPIA 2.0 indicated that high expression of BLM was significantly associated with poorer Disease-Free Survival (DFS) (*P* < 0.0001) compared to low expression of BLM (Fig. 1C).

**Table 5:** Correlations between BLM expression and clinicopathological characteristics of PCa patients

Clinicopathological characteristics	N (90)	BLM		X^2^	*P* value
		Positive (%)	Negative (%)		
Age (years)				1.327	0.185
≤ 70	49	33 (61.2)	16 (38.8)		
> 70	41	22 (53.7)	19 (17.9)		
T status				2.288	0.022
T1–T2	26	18 (69.2)	8 (30.8)		
T3–T4	64	57 (89.1)	7 (10.9)		
Gleason grade				2.743	0.006
1–3	37	25 (67.6)	12 (32.4)		
4–5	53	48 (90.6)	5 (9.4)		
N-regional lymph nodes				1.927	0.054
N0	46	32 (69.6)	18 (39.1)		
N1	44	36 (95.5)	8 (4.5)		
M-Distant metastasis				1.001	0.312
M0	88	58 (65.9)	30 (34.1)		
M1	2	2 (100)	0 (0)		

*P* value is from χ^2^-test

Moreover, the analysis of TCGA database showed a significant increase in BLM expression in PCa tissues compared to normal tissues (Fig. 1D). To validate the expression of BLM in PCa, we performed western blot analysis using four human PCa cell lines (LNCaP, PC3, 22RV1, and DU145) and human normal prostate cells (RWPE-1). Consistent with previous findings, BLM protein expression was elevated in PCa cells compared to the normal control (Fig. 1E). Subsequently, we assessed the functional impact of BLM knockdown on cell clonogenicity, viability, proliferation, invasion, and migration. The results of the colony formation assay demonstrated a significant reduction in the clonogenicity of PC3 cells upon attenuation of BLM (Fig. 1F). Additionally, the CCK-8 and EdU assays revealed a decrease in cell viability and proliferation following BLM inhibition in PC3 cells (Fig. 1G, H). Furthermore, knockdown of BLM significantly impaired the migration rate and the number of invaded PC3 cells (Fig. 1I, J). These findings highlight the notable upregulation of BLM in PCa and suggest its potential involvement in the pathogenesis of PCa.

### PARP1 was identified as a BLM promoter binding protein and negatively regulates BLM expression

**Fig. 2:**
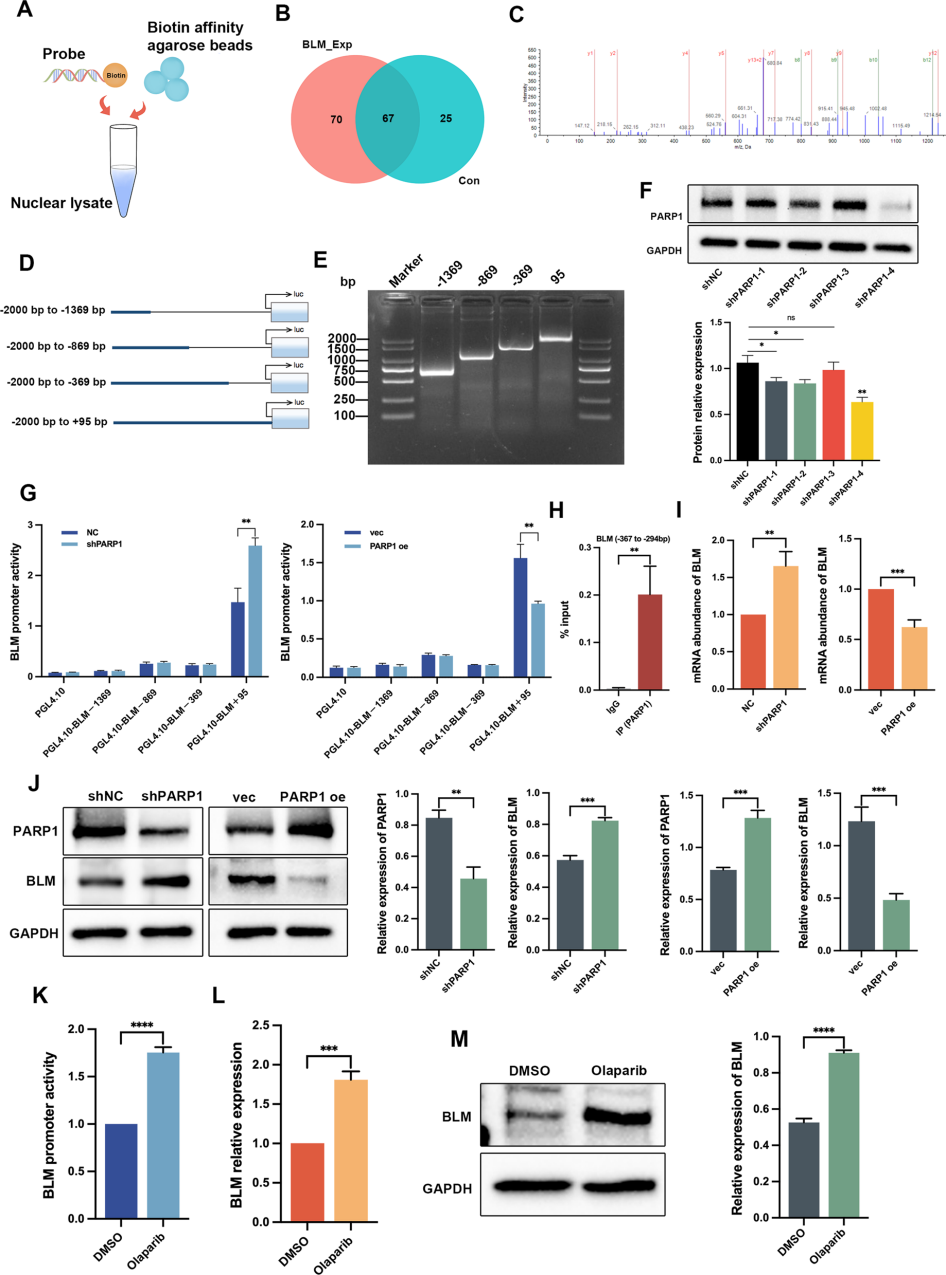
PARP1 was identified as BLM promoter-binding protein and negatively regulates BLM expression. **A** Schematic diagram illustrating the streptavidin-agarose pull-down assay used for pulling down *BLM* promoter-binding proteins in PC3 cells. **B** Venn diagram displaying the differential protein sets between BLM_Exp and Con samples. **C** Secondary MS of PARP1. **D**, **E** Fragments of the *BLM* promoter in luciferase reporter plasmids, which were detected by PCR. **F** Western blot analysis showing the efficiency of PARP1 knockdown using shRNA in PC3 cells. **G** Knockdown of PARP1 increased the activity of the pGL4.10-BLM+95 reporter plasmid, whereas overexpression of PARP1 reduced the activity of the same reporter plasmid in 293T cells. **H** ChIP-qPCR of *BLM* promoter was performed with PARP1, or control IgG antibody in PC3 cells. **I**, **J** BLM mRNA abundance, as well as PARP1 and BLM protein expression, were evaluated in PC3 cells with transient knockdown of PARP1 or overexpression of PARP1. **K**–**M** PC3 cells were treated with olaparib (45 μM) for 48 h, BLM promoter activity and mRNA abundance were measured by RT-qPCR and dual luciferase assay, respectively. BLM protein expression was detected by western blot analysis. Shown are the means ± SD from 3 experiments (*P < 0.05, **P < 0.01, ***P < 0.001, ****P < 0.0001, and “ns” indicated no significant difference)

To identify potential transcription regulators of BLM in PC3 cells, we performed a pull-down assay using a biotin-labeled DNA probe specific to the BLM promoter region. The nuclear protein-DNA complexes were captured using streptavidin-agarose beads, and the associated proteins were separated by SDS-PAGE and silver-stained (Fig. 2A). The MS identified differentially expressed proteins between the BLM_Exp and Con samples. The venn diagram revealed the identification of 162 proteins, with 67 proteins commonly identified in both samples. Specifically, 70 and 25 unique proteins were identified in the BLM_Exp and Con samples, respectively (Fig. 2B). In the BLM_Exp group, PARP1 was discovered as a candidate *BLM* promoter binding protein, and the best peptide-spectrum sequence (KGDEVDGVDEVAK) is shown in Fig. 2C. To investigate the correlation between PARP1 and BLM, we amplified truncated fragments of the *BLM* gene promoter through PCR and inserted them into the pGL4.10-basic vector, generating dual-fluorescence reporter plasmids (Fig. 2D, E). Subsequently, we constructed interference plasmids targeting PARP1, and the most efficient shPARP1-4 (hereafter referred to as shPARP1) was selected for subsequent experiments (Fig. 2F).

The dual luciferase reporter assay revealed that knockdown of PARP1 in 293T cells increased the activity of the BLM promoter fragment from − 365 bp to 95 bp, while overexpression of PARP1 decreased its activity (Fig. 2G). To validate the binding of PARP1 protein to the *BLM* promoter, ChIP-qPCR assays were performed, and it was found that PARP1 mainly bound to the region between − 367 bp and − 294 bp (Fig. 2H). Subsequently, RT-qPCR and western blot assays were conducted, which showed that interference with shPARP1 led to an upregulation of BLM mRNA abundance and protein levels, while overexpression of PARP1 had the opposite effect (Fig. 2I, J). Treatment with the PARPi olaparib also increased the promoter activity and expression of BLM (Fig. 2K–M). These findings confirmed that PARP1 bound to the *BLM* promoter and inhibited *BLM* transcription and expression in vitro.

### HSP90AB1 was identified as a regulator of BLM at the transcriptional level through its interaction with PARP1

**Fig. 3:**
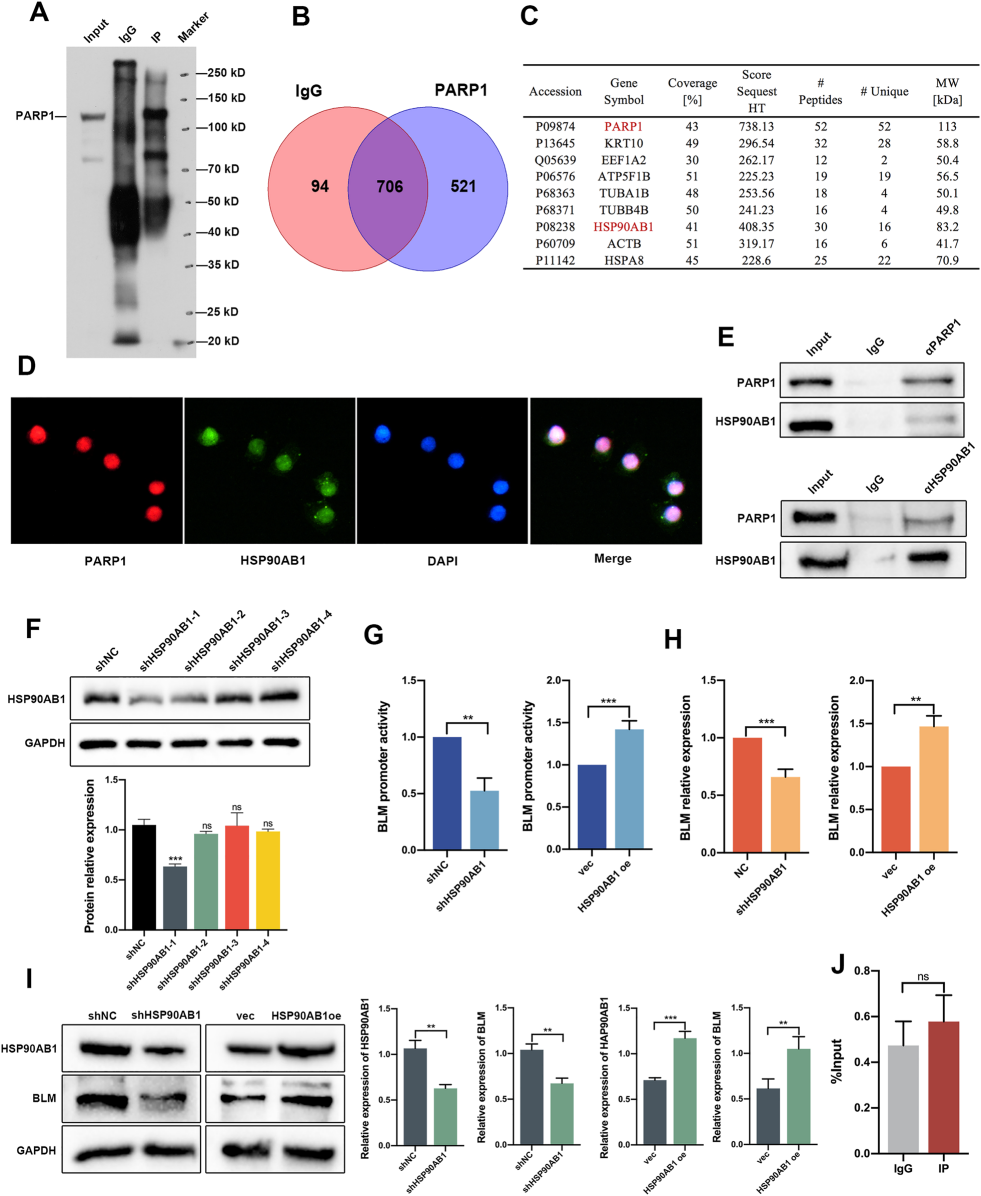

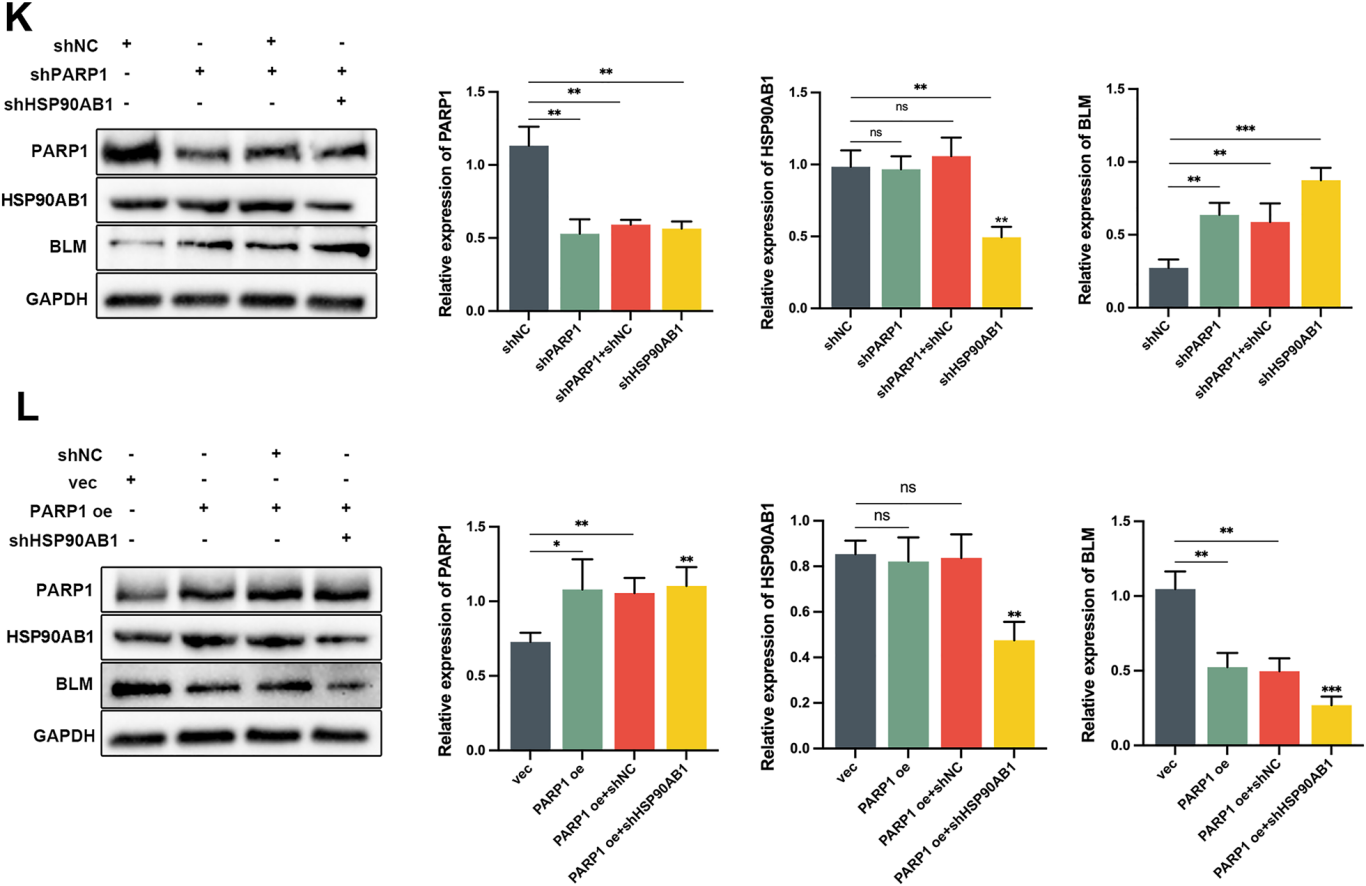
HSP90AB1 is shown to regulate BLM at the transcriptional level through its interaction with PARP1. **A**–**C** HSP90AB1 was identified as an interaction partner of PARP1 through IP and MS analyses using nuclear protein extracts from PC3 cells. **D** IF staining of PC3 cells with anti-PARP1 (red) and anti-HSP90AB1 (green) antibodies, visualizing their co-localization. Scale bar: 20 μm. **E** IP of PC3 cell lysates using anti-PARP1 (top), anti-HSP90AB1 (bottom), or control IgG antibody, followed by western blot analysis with anti-PARP1 and anti-HSP90AB1 antibodies. **F** Western blot analysis confirming the efficiency of HSP90AB1 knockdown using shRNA in PC3 cells. **G**–**I** Detection of BLM promoter activity, mRNA abundance, and protein expression of HSP90AB1 and BLM in PC3 cells transfected with shHSP90AB1 or HSP90AB1oe plasmids. **J** ChIP-qPCR of BLM promoter was performed with HSP90AB1, or control IgG antibody in PC3 cells. **K**, **L** Western blot analysis of PARP1, HSP90AB1, and BLM protein levels in PC3 cells with knockdown or overexpression of HSP90AB1, along with knockdown or overexpression of PARP1. Shown are the means ± SD from 3 experiments (*P < 0.05, **P < 0.01, ***P < 0.001, ****P < 0.0001, and “ns” indicated no significant difference)

To identify potential interaction partners of PARP1 in regulating the expression of BLM, IP was performed using nuclear protein extracts from PC3 cells (Fig. 3A). The immunoprecipitated proteins were then subjected to MS analysis. The results revealed a total of 1227 proteins in the experimental group, with 507 proteins showing differential expression compared to the control group. Among these differentially expressed proteins, 9 were identified as potential interactors with PARP1. Notably, HSP90AB1 exhibited the highest confidence score and coverage among the identified proteins (Fig. 3B, C). To further confirm the interaction between PARP1 and HSP90AB1, IF staining was performed, demonstrating the predominant co-localization of PARP1 and HSP90AB1 in the cell nuclei (Fig. 3D). To confirm the interaction between PARP1 and HSP90AB1, co-IP assays were performed using specific antibodies against PARP1 or HSP90AB1. In the anti-PARP1 IP group, the expression of HSP90AB1 was assessed (Fig. 3E, top), while in the anti-HSP90AB1 IP group, the expression of PARP1 was measured (Fig. 3E, bottom), confirming the interaction between PARP1 and HSP90AB1. Interference plasmids targeting HSP90AB1 were constructed and evaluated by western blot, with shHSP90AB1-4 (hereafter referred to as shHSP90AB1) identified as the most effective interference plasmid (Fig. 3F). Dual luciferase reporter assays were then employed to investigate the role of HSP90AB1 in *BLM* transcriptional regulation. The results showed that knockdown of HSP90AB1 inhibited *BLM* promoter activity, whereas overexpression of HSP90AB1 increased *BLM* promoter activity (Fig. 3G). Similarly, knockdown of HSP90AB1 led to a decrease in BLM mRNA abundance and protein levels, while overexpression of HSP90AB1 had the opposite effect (Fig. 3H, I). ChIP-qPCR assays demonstrated that PARP1 bound to the promoter region of BLM, whereas HSP90AB1 did not show binding to the *BLM* promoter region (Fig. 3J). Based on the interaction between HSP90AB1 and PARP1, along with their opposing effects on BLM regulation, we proposed a hypothesis that HSP90AB1 increases BLM expression by interacting with PARP1 and attenuating its negative regulatory role. To validate this hypothesis, we performed western blot assays. The results indicated that simultaneous knockdown of PARP1 and HSP90AB1 resulted in a more substantial upregulation of BLM protein levels compared to the single knockdown of PARP1 (Fig. 3K). Similarly, simultaneous overexpression of PARP1 and knockdown of HSP90AB1 led to a more pronounced downregulation of BLM protein levels compared to the single overexpression of PARP1 (Fig. 3L). These findings support the hypothesis that the interaction between HSP90AB1 and PARP1 enhances BLM expression by counteracting the negative regulatory effect of PARP1 on BLM.

### The combination of olaparib and ML216 has been found to have a superior effect in reducing the proliferation, clonogenicity, migration, and invasion of PC3 cells

**Fig. 4:**
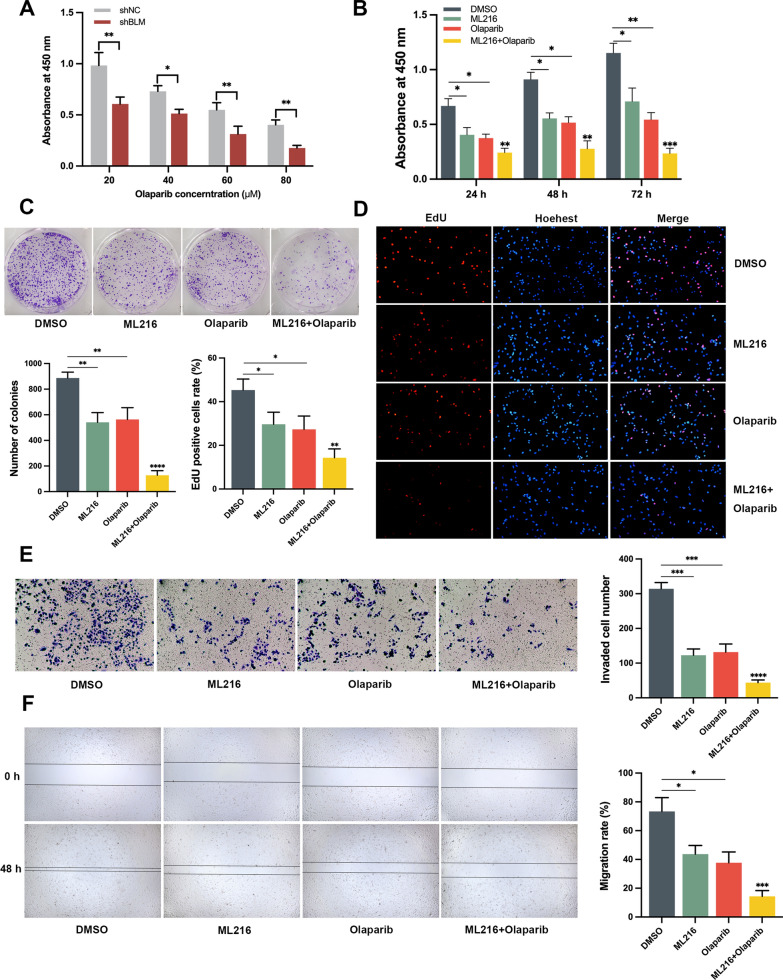
Olaparib combined with ML216 has a better effect in decreasing the proliferation and migration of PC3 cells. **A** PC3 cells with BLM knockdown were treated with increasing concentrations (20–80 μM) of olaparib for 48 h. Cell viability was assessed using the CCK-8 assay. **B**–**D** Viability, proliferation, and clonogenicity of PC3 cells were evaluated using the CCK-8, EdU, and clone formation assays, respectively, after treatment with ML216 (5 μM) and olaparib (40 μM), ML216 or olaparib monotherapy, or DMSO. Representative images and quantification are shown. Scale bars: 50 μm. **E**, **F** Invasion and migration assays were performed using the transwell and wound scratch assays, respectively, after PC3 cells treatment with ML216 (5 μM) and olaparib (40 μM), ML216 or olaparib monotherapy, or DMSO. Representative images and quantification are shown. Shown are the means ± SD from 3 experiments (*P < 0.05, **P < 0.01, ***P < 0.001, ****P < 0.0001, and “ns” indicated no significant difference)

To assess the potential role of BLM in mediating the anti-tumor sensitivity to olaparib, we silenced BLM expression using shRNA and evaluated the growth of PC3 cells in the presence of olaparib. CCK-8 assays revealed that BLM knockdown enhanced the inhibitory effect of olaparib by reducing cell viability (Fig. 4A). Furthermore, we hypothesized that the BLM helicase inhibitor ML216 might enhance the effectiveness of olaparib in PC3 cells. CCK-8 and EdU assays demonstrated that the combined treatment of ML216 and olaparib exhibited a superior inhibitory effect on PC3 cell viability and proliferation compared to treatment with ML216 or olaparib alone (Fig. 4B, C). Additionally, the combination of ML216 and olaparib displayed greater inhibitory effects on cell clonogenicity, invasion, and migration compared to monotherapy with either drug alone (Fig. 4D–F). These findings indicate that BLM inhibition enhances the anti-tumor effect of olaparib and suggest that the combination of ML216 and olaparib has a superior inhibitory effect on PC3 cells.

### Olaparib combined with ML216 has been shown to have a superior effect in enhancing total DNA damage and delaying DSB repair in PC3 cells

**Fig. 5:**
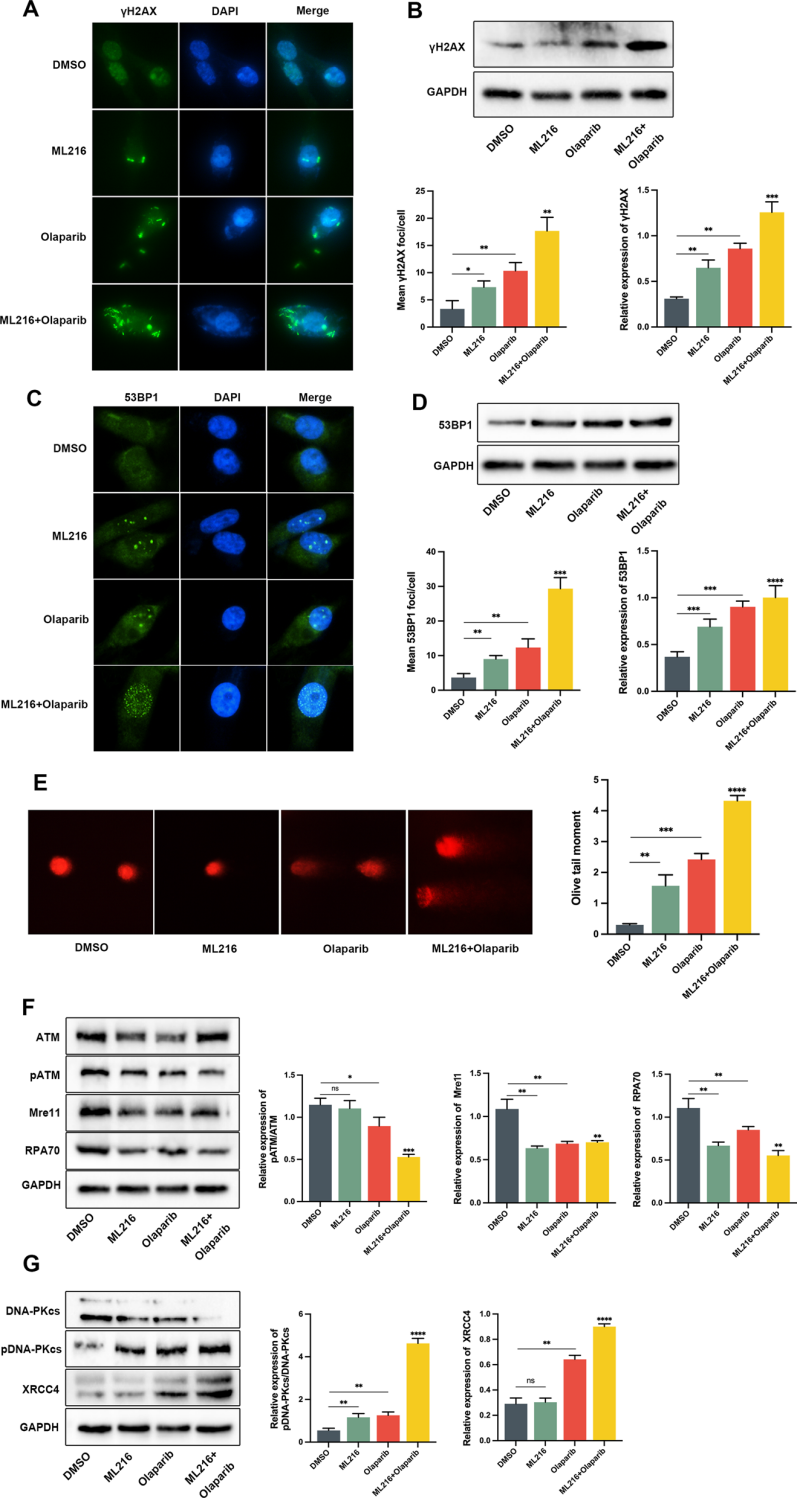
Olaparib combined with ML216 has a better effect in enhancing total DNA damage. **A**–**D** IF staining showing the expression of γH2AX and 53BP1 in PC3 cells treated with ML216 (8 μM) and olaparib (50 μM), ML216 or olaparib monotherapy, or DMSO for 48 h. Scale bars: 25 μm. **E** Comet assay of PC3 cells treated with ML216 (8 μM) and olaparib (50 μM), ML216 or olaparib monotherapy, or DMSO for 48 h.The olive tail moment was used as an index and was statistically analyzed. Scale bars: 20 μm. **F**–**G** Western blot analysis of the expression of proteins involved in the HR and NHEJ pathways in PC3 cells treated with ML216 (8 μM) and olaparib (50 μM), ML216 or olaparib monotherapy, or DMSO for 48 h. Scale bars: 20 μm. Shown are the means ± SD from 3 experiments (*P < 0.05, **P < 0.01, ***P < 0.001, ****P < 0.0001, and “ns” indicated no significant difference)

DSBs are considered the most lethal form of DNA damage. Cells have two main pathways for repairing DSBs: homologous recombination (HR) and non-homologous end joining (NHEJ) [28, 29]. The presence of γH2AX and 53BP1 foci is widely used as an indicator of DSBs [30, 31]. To evaluate the impact of olaparib and ML216 on DSBs, we assessed the formation of γH2AX and 53BP1 foci. Our results revealed a significant increase in the formation of γH2AX and 53BP1 foci in PC3 cells treated with the combination of olaparib and ML216 (Fig. 5A–C). These findings were further supported by western blot analysis (Fig. 5B–D). To investigate the extent of induced DNA damage, we employed the alkaline comet assay, with the olive tail moment serving as a measure of DNA lesions. Compared to the groups treated with each drug alone, the combination treatment of olaparib and ML216 significantly elevated the olive tail moment, indicating a greater extent of DNA damage (Fig. 5E).

To further elucidate the impact of ML216 and olaparib on DSB repair pathways, we examined key proteins involved in the HR and non-homologous end joining NHEJ pathways using western blot analysis. Our results revealed significant downregulation of key HR pathway proteins, including pATM (Ser1981), Mre11, and RPA70, in the combined drug treatment group (Fig. 5F). Conversely, key NHEJ proteins such as pDNA-PKcs (Ser2056) and XRCC4 were significantly upregulated (Fig. 5G). These findings suggest that the combination of ML216 and olaparib results in sustained impairment of DSB repair, exerting a synergistic effect by inhibiting error-free HR repair and promoting error-prone NHEJ repair in PC3 cells.

### Olaparib combined with ML216 exhibits an enhanced anti-tumor effect in PC3 xenografts in vivo

**Fig. 6:**
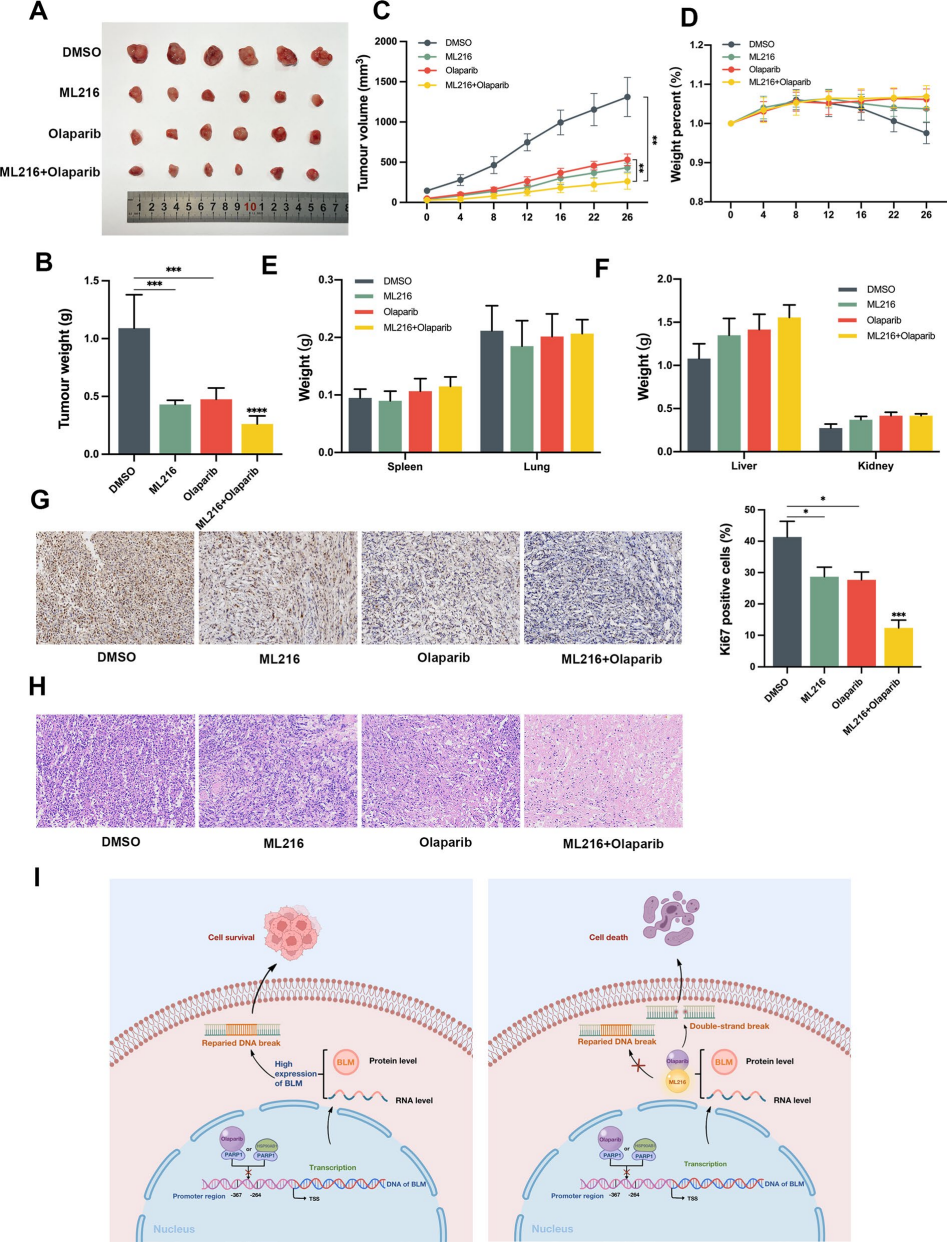
Olaparib combined with ML216 demonstrates enhanced anti-tumor effect on PC3 xenografts in vivo*.* **A** Representative images of PCa tumor xenografts from each mouse (n = 6/group). **B**, **C** The tumor weight and volume of each group were recorded and analyzed. **D** The body weights of mice were measured for 26 days. **E**, **F** The weight of main organs was measured and analyzed after sacrifice. **G** IHC of Ki-67 was performed on tumor tissue and quantified using Image software. Scale bars: 100 μm. **H** Representative images of H&E staining. Scale bars: 100 μm. **I** Schematic illustration depicting the upregulation of BLM expression by knockdown of PARP1 or interacted with HSP90AB1 and the superior anti-tumor effect observed with the combination of olaparib and ML216. Shown are the means ± SD from 3 experiments (*P < 0.05, **P < 0.01, ***P < 0.001, ****P < 0.0001, and “ns” indicated no significant difference)

Considering that inhibition of PARP1 with olaparib resulted in increased BLM expression in PC3 cells, we hypothesized that anti-BLM treatment could enhance the therapeutic efficacy of olaparib in PCa treatment in vivo. Nude mice bearing PC3-derived xenografts were treated with DMSO, olaparib (30 mg/kg/d), ML216 (15 mg/kg/d), or a combination of olaparib and ML216. On the 26th day, the tumors were excised, weighed, and subjected to IHC and H&E staining (Fig. 6A). Tumor volume and weight were measured to assess tumor growth. Compared to the DMSO group, the combination treatment of ML216 and olaparib significantly reduced the tumor volume (*P* < 0.01) and weight in PC3 xenograft mice (*P* < 0.001) (Fig. 6B, C). To assess potential toxicity in normal tissues, we monitored the body weight and weights of the main organs in mice, including the spleen, lung, liver, and kidney (Fig. 6D–F). No significant changes were observed in body weight or organ weights, indicating the absence of normal tissue toxicity. These results suggest that the combination treatment of ML216 and olaparib effectively suppressed the growth of PC3 xenograft tumors in vivo. Additionally, IHC of Ki-67 was performed to evaluate tumor proliferation in PC3 xenografts. The results revealed that the combination of ML216 and olaparib significantly reduced the expression of the Ki-67 protein in PC3 xenograft tumors (P < 0.001) (Fig. 6G), indicating a decrease in tumor cell proliferation. Histological analysis was conducted using H&E staining to observe the anti-tumor effect. In the DMSO group, the tumor tissue exhibited a dense and compact nucleus, characterized by large and deeply stained nuclei. However, treatment with ML216 and olaparib resulted in a significant reduction in tumor cell count and a looser tissue arrangement (Fig. 6H). These observations indicate the therapeutic efficacy of the combination treatment in reducing tumor cell density and altering tumor tissue organization. Overall, our findings demonstrate that the synergistic effects of ML216 and olaparib observed in cellular models can be translated to in vivo settings, supporting the potential of this combination therapy for PC3 xenografts (Fig. 6i).

## Discussion

PCa is characterized by significant genomic heterogeneity, which can arise from multiple factors, including dysregulated transcription of AR and PI3K signaling pathways, as well as DNA repair defects [32]. The AR signaling pathway plays a significant role in PCa progression, and ADT is the primary therapeutic approach for PCa. However, ADT often becomes ineffective in advanced stages of the disease due to the diverse mechanisms of various molecules. Consequently, patients treated with ADT may progress from hormone-sensitive prostate cancer to CRPC [33]. Therefore, there is increasing recognition of the importance of a personalized treatment approach that takes into account the patient’s specific genetic profile in managing PCa [34]. Mutations in DNA helicase genes have been identified as factors that increase an individual's susceptibility to developing cancer [35, 36]. Specifically, RECQ helicases, including BLM, WRN, and RECQL4, are associated with various hereditary disorders characterized by cancer. These helicases play a role in enabling cancer cells to withstand replicative stress and facilitate uncontrolled growth and division, particularly in situations where cells accumulate replicative lesions at a high rate. Excitingly, certain DNA helicases have gained attention as promising targets for anti-cancer therapy [37]. Among them, BLM stands out as a crucial genome stabilizer that plays a vital role in regulating DNA replication, recombination, and DSB repair through both homologous and non-homologous pathways. Mutations in the BLM gene can lead to significant growth defects and heightened susceptibility to cancers and other diseases [38]. Previous studies have demonstrated a significant association between BLM and PCa, suggesting its potential as a biomarker for targeted therapy in PCa [39, 40]. However, the upstream regulatory mechanisms governing BLM in PCa are still not well understood.

In this study, we first evaluated the expression levels of BLM in both PCa tumor tissues and cell lines. Our results demonstrated a significant increase in BLM expression in PCa. Subsequently, we investigated the role of BLM in cell proliferation and migration, revealing its critical involvement in these processes. These findings suggest that the overexpression of BLM in PCa may contribute to tumor promotion by facilitating cell proliferation and migration. Considering that regulatory events often occur at gene promoters, we aimed to identify the endogenous transcriptional regulators of BLM in PC3 cells.

To identify nuclear proteins specifically binding to the *BLM* promoter, we employed a streptavidin-agarose pull-down assay combined with MS, using a biotin-labeled BLM promoter probe in PC3 cells. Among the identified candidates, PARP1 drew our attention most, prompting further investigation. PARP1 is involved in various cellular and biological processes, including DNA damage response (DDR), base excision repair (BER), and DSB pathways. Additionally, PARP1 plays critical roles in cancer biology, including genome maintenance, replication, transcription, and chromatin remodeling [41]. Although PARP1 was initially recognized for its significant involvement in DNA repair and genomic maintenance, recent studies have revealed that PARP1 also plays diverse roles in transcriptional regulation [42, 43]. To elucidate the molecular mechanisms underlying the interaction between BLM and PARP1, we performed a luciferase assay. The results of our study demonstrated that knockdown of PARP1 led to an increase in promoter activity, while overexpression of PARP1 resulted in a decrease in promoter activity, specifically on the pGL4.1-BLM+95 reporter plasmid. ChIP-qPCR assays were performed to demonstrate that PARP1 primarily binds to the BLM promoter region within the − 367 to − 294 bp range. Moreover, both mRNA abundance and protein levels of BLM were found to be inhibited by PARP1 overexpression and increased by PARP1 knockdown. Similarly, when PC3 cells were treated with olaparib, a significant increase in BLM promoter activity, mRNA, and protein levels was observed. Our experiments in PC3 cells confirmed that PARP1 binds to the BLM promoter, resulting in decreased activity and reduced expression of both BLM mRNA and protein. These findings suggest that PARP1 functions as an endogenous negative transcriptional regulator of BLM.

Notably, our findings revealed that HSP90AB1 plays a regulatory role in BLM transcription through its interaction with PARP1. Knockdown of HSP90AB1 led to decreased *BLM* promoter activity, mRNA expression, and protein levels, while overexpression of HSP90AB1 had the opposite effect. These results suggest that HSP90AB1 enhances *BLM* transcription, which counteracts the negative regulation by PARP1. Subsequently, ChIP-qPCR assays were performed to verify whether HSP90AB1 binds to the *BLM* promoter region or not. However, the results indicated that only PARP1, and not HSP90AB1, associates with the promoter region of *BLM*. IF staining revealed that PARP1 and HSP90AB1 were predominantly co-localized in the nucleus of cells, and their interaction was further confirmed through co-IP analysis. Western blot analysis demonstrated an upregulation of BLM protein levels upon knockdown of PARP1. Furthermore, when both PARP1 and HSP90AB1 were knocked down, BLM protein levels were even more significantly upregulated. In contrast, overexpression of PARP1 alone resulted in a downregulation of BLM protein levels. However, when PARP1 overexpression was combined with HSP90AB1 knockdown, the downregulation of BLM caused by PARP1 overexpression was alleviated. Therefore, we concluded that HSP90AB1 upregulates BLM transcription by interacting with PARP1 and counteracting its negative regulation of BLM.

Olaparib, a PARPi, has received approval for the treatment of metastatic breast cancer and mCRPC patients with genetic mutations in HR repair genes. It has been shown to offer significant clinical benefits in these patient populations [44]. Approximately 30% of advanced CRPC patients have deficiencies in HRR genes, either in their germline or somatic DNA. These deficiencies make them sensitive to treatment with PARP inhibitors. The TOPARP-A and TOPARP-B clinical trials have shown significant progress in the treatment of mCRPC patients with HRR deficiencies using PARP inhibitors [45, 46]. Similar to other targeted therapies, the development of drug resistance is an inevitable challenge that hinders the clinical effectiveness of PARP inhibitors in patients with mCRPC [47]. Based on the previous findings, which demonstrated that knockdown of PARP1 or inhibition of PARP1 using olaparib results in the upregulation of BLM activity, mRNA abundance, and protein levels, we hypothesized that the inhibition of BLM by ML216 could enhance the anti-tumor effectiveness of olaparib against PCa. Given the crucial role of BLM in maintaining genomic stability, this combination therapy approach holds the potential to augment the overall therapeutic efficacy against PCa. The results of the study demonstrated that the combination treatment of ML216 and olaparib exhibited significantly stronger inhibitory effects on PC3 cell proliferation compared to either ML216 or olaparib alone, both in vitro and in vivo. Moreover, the combination treatment led to an increase in DSB markers and DNA damage, as evidenced by enhanced formation of γH2AX and 53BP1 foci and increased tail length. These findings indicate that the combination of ML216 and olaparib synergistically enhances DSBs and DNA damage. In contrast to the highly conserved and error-free HR repair pathway, the NHEJ pathway is more prone to errors, leading to genomic instability [48, 49]. By inhibiting PARP1, olaparib effectively impedes the repair process of DNA lesions, specifically DSBs, resulting in the accumulation of unrepaired DNA damage. HR repair is a precise and error-free mechanism that employs a homologous DNA template to accurately mend DSBs. Previous investigations have demonstrated the involvement of BLM in stimulating the strand exchange activity of Rad51, a key player in HR repair. Hence, the inhibition of BLM by ML216 disrupts the formation of RAD51 filaments, thereby impairing the HR repair pathway [16, 50, 51]. Conversely, NHEJ repair represents an alternative mechanism for DSB repair that directly joins fragmented DNA ends, often resulting in insertions, deletions, or other genetic alterations. Our findings suggest that the combination of ML216 and olaparib augments reliance on the NHEJ repair pathway, as the error-free HR pathway is inhibited.

## Conclusions

This study provides evidence that BLM is highly expressed in PCa and promotes the proliferation of PC3 cells. Additionally, we have identified PARP1 as a negative regulator of BLM, and HSP90AB1 as a facilitator of BLM transcription mediated by PARP1. These findings establish a solid basis for the combination therapy that targets BLM inhibition and PARP inhibition in the treatment of PCa.

## Data Availability

The data used to support the findings of this study are included within the article. Additional data and materials are available from the corresponding author upon reasonable request.

## Abbreviations

PCa: Prostate cancer
BLM: Bloom syndrome protein
PARP1: Poly (ADP-ribose) polymerase 1
HSP90AB1: Heat shock protein alpha family class B
Co-IP: Co-immunoprecipitation
ChIP-qPCR: Chromatin Immnoprecipitation-qPCR
HR: Homologous repair
NHEJ: Non-homologous end-joining
DSB: Double strand breaks
